# Novel Pathogenic Variant Confirms the Association of *REST* and Jones Syndrome

**DOI:** 10.1111/cge.70017

**Published:** 2025-07-02

**Authors:** Valentina Lodato, Massimo Galli, Giacomo D'Angeli, Irene Bottillo, Luca Celli, Rosaria Turchetta, Andrea Colizza, Francesca Gianno, Biagio Palmisano, Francesca Romana Federici Stanganelli, Maria Rita Bianco, Daniela Messineo, Eugenia Allegra, Paola Grammatico, Mara Riminucci, Alessandro Corsi

**Affiliations:** ^1^ Department of Experimental Medicine, Division of Medical Genetics San Camillo‐Forlanini Hospital Rome Italy; ^2^ Department of Odontostomatological Science and Maxillo‐Facial Surgery Sapienza University of Rome Rome Italy; ^3^ Department of Sense Organs Sapienza University of Rome Rome Italy; ^4^ Department of Radiology, Oncology and Anatomical Pathology Sapienza University of Rome Rome Italy; ^5^ Department of Molecular Medicine Sapienza University of Rome Rome Italy; ^6^ Department of Health Science University of Catanzaro Catanzaro Italy

**Keywords:** exome sequencing, gingival fibromatosis, Jones syndrome, *REST*, sensorineural hearing loss

## Abstract

Jones syndrome (JS) is an ultra‐rare autosomal dominant condition characterized by gingival fibromatosis and progressive sensorineural hearing loss. It has been recently demonstrated in members of a Finnish family to co‐segregate with heterozygosity for a frameshift variant in the fifth and last exon of the repressor element 1‐silencing transcription factor gene (*REST*). Here, we report the first Italian family in which JS was diagnosed in the proband, a 38‐year‐old woman, and in her mother. Exome Sequencing identified in both, but not in clinically unaffected members of the family (i.e., a sister and the brother of the proband), the heterozygous pathogenic variant c.2645T>G (p.Leu882*) in exon‐5 of the *REST* gene. This study confirms that exon‐5 *REST* variants cause JS.

## Graphical abstract

Jones syndrome (JS) is an ultra‐rare condition characterized by gingival fibromatosis and progressive sensorineural hearing loss. It has been associated with a pathogenic *REST* exon‐5 variant (c.2670_2673del) in a Finnish family. We describe the first Italian family with JS in which a novel pathogenic *REST* exon‐5 variant (c.2645T>G) was identified.

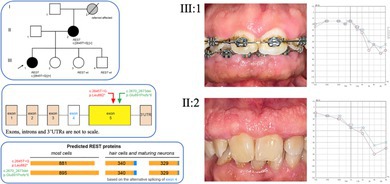

## 1 Introduction

Jones Syndrome (JS, OMIM %135550) is an autosomal dominant condition characterized by gingival fibromatosis (GF) and progressive sensorineural hearing loss (SNHL). Less than 20 cases have been reported [1]. A pathogenic variant in the fifth, and last, exon of the repressor element 1‐silencing transcription factor gene (*REST*, OMIM *600571) segregating with the JS phenotype has been recently reported in members of a Finnish family [1]. Pathogenic variants of *REST* have also been associated with hereditary isolated/non‐syndromic GF [2, 3, 4] and SNHL [5, 6, 7] and with susceptibility to Wilms tumor [8].

We report here the first Italian family with JS. Through Exome Sequencing (ES), we identified a new heterozygous pathogenic exon‐5 *REST* variant segregating with the phenotype.

## 2 Methods

### 2.1 Clinical Data

Affected and unaffected members of an Italian family were included in this study. All the clinic‐pathologic and genetic investigations were conducted in accordance with the Declaration of Helsinki and its later amendments. The study was approved by the San Camillo‐Forlanini Hospital Ethics Board. Written informed consent was obtained from all the participants.

### 2.2 Genetic Analysis

**FIGURE 1:**
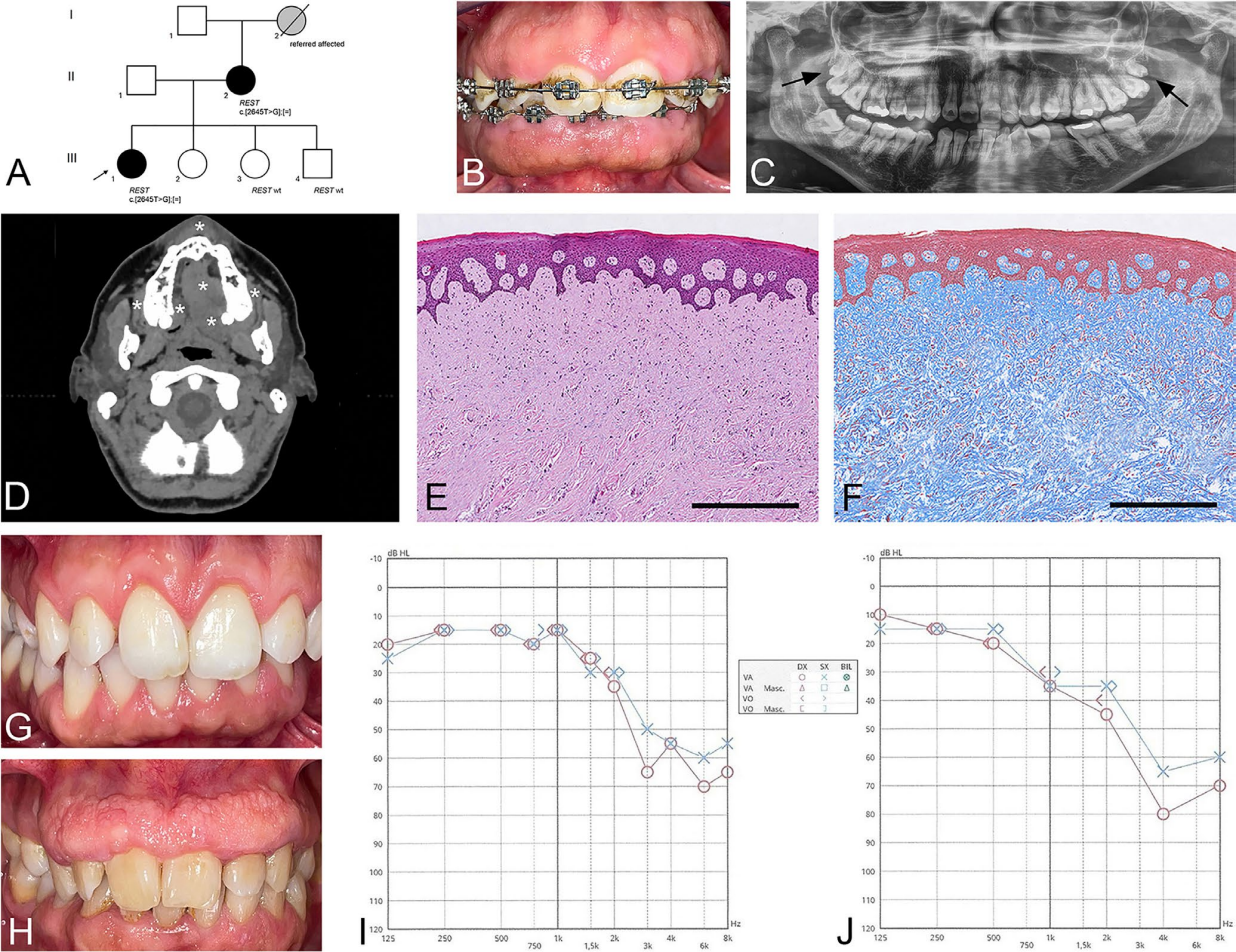
(A) Pedigree of the family in which the proband is identified by the arrow and the result of molecular analysis reported. (Panels B–G and I) Patient‐1. (Panels H and J) Patient‐2. Clinical presentation, OPG, and CT of patient‐1 are shown in B, C, and D respectively. In the OPG, supernumerary teeth are identified by arrows. In the axial CT image, the lesional tissue is identified by asterisks. Histological images of the gingival lesion are shown in E (Haematoxylin‐Eosin) and F (Masson's Trichrome). Clinical presentation 18 months after surgery is shown in G. (H) Clinical view of patient‐2. Audiograms of patient‐1 and patient‐2 are shown in I and J respectively. Bar in (E and F) 500 μm.

ES was performed on the genomic‐DNA extracted from peripheral blood of four members of the family, including the proband (III:1), her mother (II:2), one of her sisters (III:3), and the brother (III:4) (Figure 1A). Procedure details are included in the Supporting Information.

## 3 Results

### 3.1 Clinical Data

The proband, a 38‐year‐old woman (patient‐1), presented in 2022 with gingival and palatal overgrowth that began during adolescence. She denied pharmacological treatments and referred that her mother (patient‐2, 63‐year‐old) and maternal grandmother (I:2, passed away in 2023) presented similar findings. Oral examination revealed marked tissue hypertrophy throughout the entire oral cavity (Figure 1B). She showed class I canine and molar relationships bilaterally, transverse maxillary deficiency without crossbite, and a deep bite with zero overjet. Cephalometric analysis revealed a convex profile with skeletal hyper‐divergence. Extraoral examination failed to reveal changes in skin, hair, and osteo‐muscular system. Orthopantomography (OPG) revealed bilateral supernumerary teeth after wisdom teeth in the maxilla (Figure 1C). Computed Tomography (CT) highlighted the extension and boundaries of the lesion (Figure 1D). Electro‐surgical resection of the hypertrophic tissue was performed. Histological examination of the excised samples revealed hypocellular, hypovascularized, and dense sub‐epithelial connective tissue consistent with GF (Figure 1E,F). The postoperative course was uneventful, and proper healing was observed at follow‐up (Figure 1G).

Based on the familiarity of the gingival overgrowth and genetic heterogeneity of GF [9], ES was performed in patient‐1 and patient‐2. Following the results of the genetic analyses, patient‐2 was visited and both underwent pure tone audiometry (PTA). Oral examination confirmed the gingival overgrowth in patient‐2 (Figure 1H). A surgical treatment was proposed, but it was refused. PTA revealed bilateral SNHL of moderate degree on high frequencies in patient‐1 (Figure 1I) and bilateral SNHL of moderately severe degree on high frequencies in patient‐2 (Figure 1J).

Clinical examination, PTA and ES were also performed in a 33‐year‐old sister and in the 24‐year‐old brother of the proband (Figure S1). Mild gingival hypertrophy, clinically interpreted as a consequence of periodontal disease related to his smoking habit and poor oral hygiene, was observed in the brother. PTA was negative in both.

Interrogation of the patients also revealed that hearing impairment was present in the maternal grandmother and that none of them, including the other sister of the proband (III:2, currently 39‐year‐old), for whom gingival overgrowth and hearing impairment were denied, had a history of Wilms's and other malignant tumors.

### 3.2 Genetic Analysis

**FIGURE 2:**
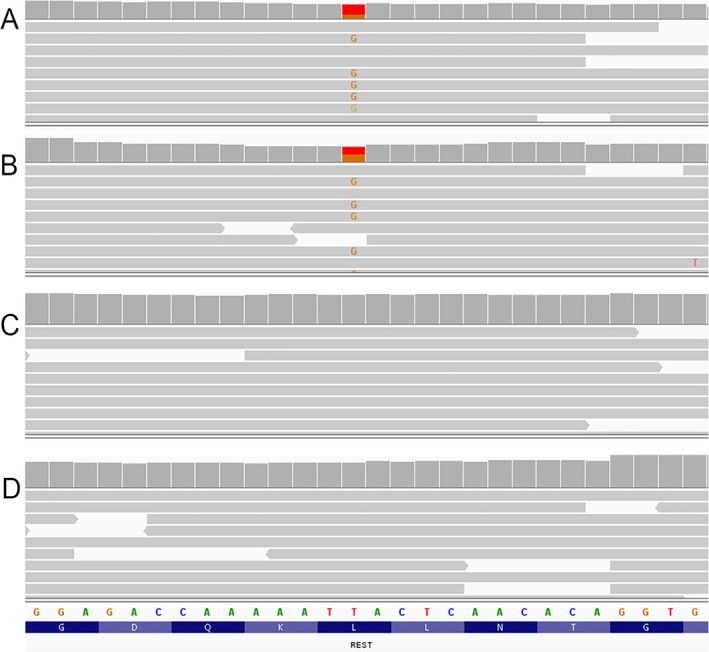
Molecular characterization of the *REST* gene in patient‐1, in patient‐2, in the sister (III:3) and in the brother (III:4) of the proband is shown in A, B, C, and D respectively. Sequencing reads show the presence of the heterozygous *REST*: c.2645T>G (p.Leu882*) variant only in patient‐1 and patient‐2.

ES analysis identified a heterozygous exon‐5 *REST* variant, c.2645T>G (p.Leu882*), in patient‐1 and patient‐2 (Figure 2A,B) but not in the proband's sister and brother (Figure 2C,D). The variant was classified as pathogenic according to the ACMG/AMP criteria [10]: PVS1_strong (nonsense variant not predicted to undergo nonsense mediated decay, but removing > 10% of the protein sequence); PM2_supporting (variant absent in GnomAD 4.1 population database); PP4_strong (patient's phenotype and family history specific for JS); BS4_supporting (lack of segregation in a family member, i.e., III:4). Although none of the investigated subjects carried any other likely pathogenic variant in the genes with established clinical relevance for GF (Supporting Information), patient‐1 and patient‐2 were both carriers of the variant c.1202_1205delTATT in *CD36* (Table S1), a recently reported novel GF candidate gene [4]. This variant was absent in cases III:3 and III:4 and was classified as a variant of unknown significance according to the PVS1_strong (nonsense variant not predicted to undergo nonsense mediated decay, but removing > 10% of the protein sequence) ACMG/AMP criteria [10].

## 4 Discussion

This report describes the first Italian family with JS and the second worldwide one in which JS associates with an exon‐5 *REST* variant.

JS is an ultra‐rare condition in which GF typically precedes SNHL [1]. Indeed, the latter starts at high frequencies thus requiring PTA to be detected in the early stage. However, additional findings including hirsutism, undescended testis, hypermobility of the distal phalanges, and supernumerary teeth, the last of which was also observed in the proband, have been reported in patients with JS, supporting its clinical variability [1, 11].

*REST* encodes a transcriptional repressor that regulates gene expression by binding and recruiting co‐repressors to repressor element‐1 sites in many genes [12]. This protein complex forms and represses REST target genes in most cell types except neurons and pancreatic β‐cells, in which *REST* expression is minimal due to transcriptional repression, and inner ear hair cells, in which regulated alternative splicing of the REST pre‐mRNA prevents expression of an active REST protein [6, 12, 13, 14, 15].

JS has been recently associated with a pathogenic variant of *REST*, c.2670_2673del (p.Glu891Profs*6), in three members (father and two daughters) of a Finnish family [1]. This variant shifts the translational reading frame in exon‐5 of *REST* such that the encoded protein lacks one of the nine zinc finger domains and one of the two repressor domains that mediate the recruitment of co‐repressors [1, 12]. The heterozygous variant c.2645T>G (p.Leu882*) detected in our family introduces a premature translation stop codon. *REST* variants described to date in association with hereditary isolated/non‐syndromic GF and SNHL and with JS are reported in Table 1. Notably, all the variants involve exon‐5 of *REST* except one. The exception is Deafness Autosomal Dominant 27 (DFNA27) [5, 6, 12] in which the variant determines a novel splice acceptor site in intron‐3 preventing the alternative splicing of exon‐4 necessary for the inactivation of REST in the inner ear hair cells, and the SNHL is uniform across all audible frequencies and not, as in JS, limited to high frequencies in the early stage [1, 12].

**TABLE 1:** *REST* constitutional variants associated with JS and with hereditary isolated/non‐syndromic GF and SNHL.

Variant	Exon/intron	Phenotype	References
c.2645T>G (p.Leu882*)	Exon 5	JS	This report
c.2670_2673del (p.Glu891Profs*6)			[1]
c.2865_2866delAA (p.Asn958Serfs*9) c.1310T>A (p.Leu437*) c.2413delC (p.Leu805Phefs*38)		GF	[2]
c.2449C>T, (p. Arg817*) c.2771_2793dup (p.Glu932Lysfs*3)			[3]
c.1493_1494del (p.Glu498Glyfs*17)			[4]
c.1244G>C (p.Cys415Ser)		SNHL	[7]
c.983‐2247C>G	Intron 3		[6]

*Note:* Isoform reference is *REST*: NM_005612.5.

The mechanisms by which exon‐5 *REST* variants impact gingival mucosa and inner ear have not been conclusively defined. The c.2645T>G (p.Leu882*) variant leads to protein truncation predicted to escape nonsense mediated decay but removes > 10% of the protein sequence. If so, both GF and SNHL could be related to a loss‐of‐function mechanism. A gain‐of‐function effect seems unlike as studies in mice reported that downregulation of *REST* contributed to hearing impairment [16]. A dominant negative effect has been suggested by Chen et al. [3] who demonstrated that truncated REST proteins impair the repressor activity of co‐expressed wild‐type REST, competing with it for DNA binding.

REST is known to play different functions in development and diseases [17, 18]. However, REST target genes have been only partially identified. They include genes involved in signaling pathways (i.e., TGF‐β/Smad) regulating the synthesis of extra‐cellular matrix components, especially type‐I collagen [19]. Therefore, up‐regulation of these pathways resulting from reduction of the repressor activity of REST could contribute to the increase in the synthesis of the type‐I collagen enriched extra‐cellular matrix that characterizes GF [4, 9]. Moreover, genomic deletion of exon‐4 of *REST* in mice reduces the expression of many hearing‐related genes [6]. However, further studies are needed to elucidate the molecular mechanisms that make gingival mucosa and inner ear so sensitive to exon‐5 *REST* variants.

Interestingly, patient‐1 and patient‐2 were also found to carry a variant of unknown significance in *CD36*, a gene recently proposed as a novel candidate for GF [4]. Even if the role of CD36 in GF has not been demonstrated to date [4], we cannot exclude that the c.1202_1205delTATT variant could act as a disease modifier in our patients [20].

For its rarity, maxillofacial surgeons, dentists, and otolaryngologists might not be familiar with JS. GF and SNHL may be mild, age‐related, or even non‐penetrant so that patients with hereditary isolated/non‐syndromic GF or SNHL and exon‐5 *REST* variants may be affected by JS. It is well established that a thorough clinical and genetic investigation is needed in a young patient with GF [9]. We feel that PTA must be included in this work‐up, especially when exon‐5 *REST* variants are detected. Early diagnosis of JS may allow for timely treatments to reduce disease severity and improve the patients' social life.

## Author Contributions

Conceptualization and writing original draft: Valentina Lodato and Alessandro Corsi. Writing review and editing: Valentina Lodato, Massimo Galli, Giacomo D'Angeli, Irene Bottillo, Luca Celli, Rosaria Turchetta, Andrea Colizza, Francesca Gianno, Biagio Palmisano, Francesca Romana Federici Stanganelli, Maria Rita Bianco, Daniela Messineo, Eugenia Allegra, Paola Grammatico, Mara Riminucci, and Alessandro Corsi. All authors revised the paper critically for intellectual content, approved the final version, and agreed to be accountable for the work.

## Conflicts of Interest

The authors declare no conflicts of interest.

## Supporting information


Data S1.

## Data Availability

The data that support the findings of this study are available from the corresponding author upon reasonable request.
