# Supplementary material for: Novel Pathogenic Variant Confirms the Association of REST and Jones Syndrome
Source: Clin Genet. 2025 Jul 2;109(1):199–203. doi: 10.1111/cge.70017 (PMC12674978; doi:10.1111/cge.70017)
Supplement: Supplementary file 1 — Data S1. [file CGE-109-199-s001.docx]

**SUPPLEMENTARY MATERIAL**

**Novel pathogenic variant confirms the association of *REST* and Jones Syndrome**

Valentina Lodato^1^, Massimo Galli^2^, Giacomo D’Angeli^2^, Irene Bottillo^1^, Luca Celli^1^, Rosaria Turchetta^3^,

Andrea Colizza^3^, Francesca Gianno^4^, Biagio Palmisano^5^, Francesca Romana Federici Stanganelli^2^, Maria Rita Bianco^6^,

Daniela Messineo^4^, Eugenia Allegra^6^, Paola Grammatico^1^, Mara Riminucci^5^, Alessandro Corsi^5^

^1^ Department of Experimental Medicine, Division of Medical Genetics, San Camillo-Forlanini Hospital, Sapienza University, Rome, Italy.

^2^ Department of Odontostomatological Science and Maxillo-Facial Surgery, Sapienza University, Rome, Italy.

^3^ Department of Sense Organs, Sapienza University, Rome, Italy.

^4^ Department of Radiology, Oncology and Anatomical Pathology, Sapienza University, Rome, Italy.

^5^ Department of Molecular Medicine, Sapienza University, Rome, Italy.

^6^ Department of Health Science, University of Catanzaro, Catanzaro, Italy.

**Corresponding author**

Prof. Alessandro Corsi

Department of Molecular Medicine

Sapienza University

e-mail: alessandro.corsi@uniroma1.it

***Exome Sequencing***

Exome Sequencing was performed on the genomic-DNA extracted from peripheral through the Nextera Exome kit on the NextSeq2000 sequencer (Illumina, San Diego, CA, USA). Sequencing reads were aligned to the human reference genome (UCSC hg19) and variant calling was performed by GATK (v1.6-23-gf0210b3). The DNA variants were annotated by eVai (v.3.0) (EnGenome) and filtered by MAF (Minor Allele Frequency in GnomAD4.1 population database) <0.01,and by the genes to date associated to the Human Phenotype Ontology HP: 0000169 Gingival Fibromatosis [1] including *ABCA5, ANTXR2, ATP6V1B2, CDKN1A, CDKN1B, CDKN2B, CDKN2C, DHCR24, ELMO2, FAM20A, FAM20C, IFNG, KCNH1, KCNN3, MEN1, NOTCH3, PDGFRB, REST, SOS1, TSC1* and *TSC2*. Variants were categorized into five classes (pathogenic, likely pathogenic, VUS, likely benign, and benign) as defined by the Human Phenotype Ontology database according to ACMG/AMP criteria [2].

***Clinical data***


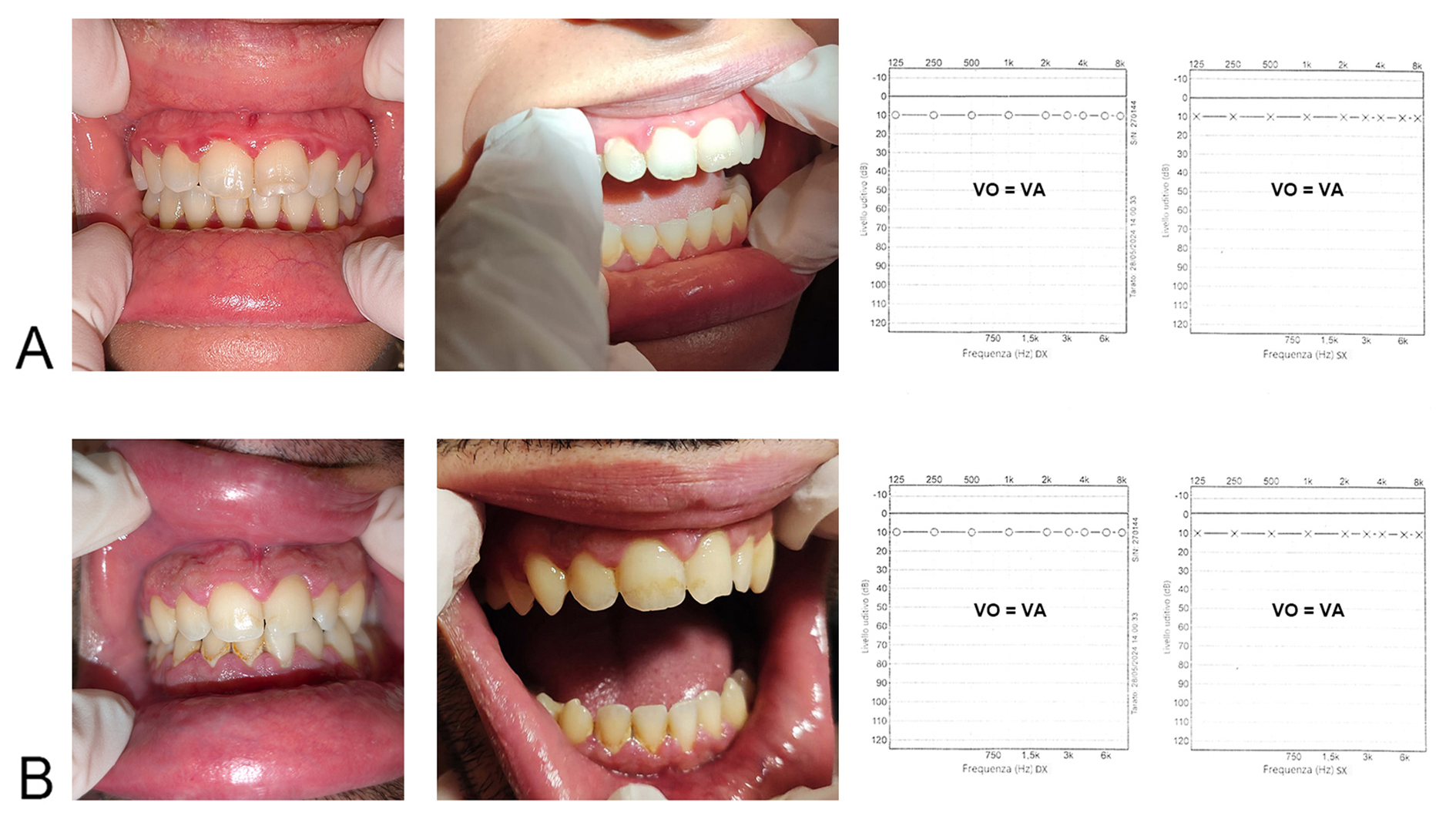


**Figure S1.** Oral examinations and audiograms of the 33-year-old sister (III:3) and of the 24-year-old brother (III:4) of the proband are shown in panels A and B respectively.­­­ In the audiograms, VO and VA are for Bone Conduction and Air Conduction respectively.

***Genetic analysis***

The GeneMatcher platform was used to identify additional families with pathogenic *REST* variants and the JS phenotype [3]. However, additional families with this genotype/phenotype match were not reported.

**Table S1. Relevant filtered variants co-segregating in the *patient-1* and *patient-2.***

| **Family**  **model** | ***Gene***  **(Chr)** | **position (hg19)** | **Ref** | **Alt** | **Transcript** | **Variant** | **Allele** | **Inheritance** | **OMIM**  **disease** | **ACMG/AMP**  **classification** |
| --- | --- | --- | --- | --- | --- | --- | --- | --- | --- | --- |
| AD | *REST*  *(*4) | 57797669 | T | G | NM_005612.5 | c.2645T>G | Het | AD | #617626 | P |
| AD | *CD36*  *(*7) | 80302673 | TATT | - | NM_001001548.2 | c.1202_1205delTATT | Het | AR | #608404 | VUS |
| AD | *SBF1*  (22) | 50886831 | G | A | NM_002972.4 | c.5194C>T | Het | AR | #615284 | VUS |

Chr: chromosome; Het: heterozygous; AD: autosomal dominant; AR: autosomal recessive; P: Pathogenic; VUS: variant of unknown significance.

**Exome sequencing raw data parameters**

*Patient-1 (III:1)*: Aligned reads in target region: 76,292,976; Average coverage over target region: 183.28 x; Uniformity of coverage: 94.44%; % of target region with coverage ≥20x: 95.86; Total number of variants: 40,960.

*Patient-2 (II:2)*: Aligned reads in target region: 74,405,007; Average coverage over target region: 178.40x; Uniformity of coverage: 94.54%; % of target region with coverage ≥20x: 95.90; Total number of variants: 41,195.

**Cited references**

1. https://hpo.jax.org/browse/term/HP:0000169 (last accessed 23 May 2025).

2. Richards S, Aziz N, Bale S et al. Standards and guidelines for the interpretation of sequence variants: a joint consensus recommendation of the American College of Medical Genetics and Genomics and the Association for Molecular Pathology. Genet Med. 2015;17:405-24.

3. Sobreira N, Schiettecatte F, Valle D, Hamosh A. GeneMatcher: a matching tool for connecting investigators with an interest in the same gene. Hum Mutat. 2015;36:928-930.
